# Reciprocal Regulation of MAGED2 and HIF-1α Augments Their Expression under Hypoxia: Role of cAMP and PKA Type II

**DOI:** 10.3390/cells11213424

**Published:** 2022-10-29

**Authors:** Elie Seaayfan, Sadiq Nasrah, Lea Quell, Aline Radi, Maja Kleim, Ralph T. Schermuly, Stefanie Weber, Kamel Laghmani, Martin Kömhoff

**Affiliations:** 1 University Children’s Hospital, Philipps University, 35043 Marburg, Germany; 2 Department of Internal Medicine, Member of the German Center for Lung Research (DZL), Excellence Cluster Cardio-Pulmonary Institute (CPI), Justus-Liebig-University, 35392 Giessen, Germany; 3 Centre de Recherche des Cordeliers, Sorbonne Université, Inserm, Université de Paris, CNRS, ERL8228, F-75006 Paris, France

**Keywords:** MAGED2, hypoxia, HIF-1α, GalphaS, PKA type II, Bartter

## Abstract

Hypoxia stabilizes the transcription factor HIF-1α, which promotes the transcription of many genes essential to adapt to reduced oxygen levels. Besides proline hydroxylation, expression of HIF-1α is also regulated by a range of other posttranslational modifications including phosphorylation by cAMP-dependent protein kinase A (PKA), which stabilizes HIF-1α. We recently demonstrated that MAGED2 is required for cAMP generation under hypoxia and proposed that this regulation may explain the transient nature of antenatal Bartter syndrome (aBS) due to *MAGED2* mutations. Consequently, we sought to determine whether hypoxic induction of HIF-1α requires also MAGED2. In HEK293 and HeLa cells, MAGED2 knock-down impaired maximal induction of HIF-1α under physical hypoxia as evidenced by time-course experiments, which showed a signification reduction of HIF-1α upon MAGED2 depletion. Similarly, using cobalt chloride to induce HIF-1α, MAGED2 depletion impaired its appropriate induction. Given the known effect of the cAMP/PKA pathway on the hypoxic induction of HIF-1α, we sought to rescue impaired HIF-1α induction with isoproterenol and forskolin acting upstream and downstream of Gαs, respectively. Importantly, while forskolin induced HIF-1α above control levels in MAGED2-depleted cells, isoproterenol had no effect. To further delineate which PKA subtype is involved, we analyzed the effect of two PKA inhibitors and identified that PKA type II regulates HIF-1α. Interestingly, MAGED2 mRNA and protein were also increased under hypoxia by a cAMP mimetic. Moreover, MAGED2 protein expression also required HIF-1α. Thus, our data provide evidence for reciprocal regulation of MAGED2 and HIF-1α under hypoxia, revealing therefore a new regulatory mechanism that may further explain the transient nature of aBS caused by MAGED2 mutations.

## 1. Introduction

During gestation, the fetus is exposed to a very low arterial O_2_ tension (~25 mmHg) as compared with that of adults (~95 mmHg) [1]. Despite low oxygen levels, salt and water reabsorption in the renal medulla, which has a much lower supply of oxygen compared with the renal cortex [2,3,4,5], is very effective in the fetus, as evidenced by loss of function mutations in NKCC2 (which is expressed in the renal medulla and reabsorbs about 30% of filtered sodium) causing profound renal salt wasting [6]: The latter causes excessive amniotic fluid production and hence polyhydramnios leading to preterm birth and severely increased risk of intraventricular hemorrhage.

We and others have recently shown, that truncating mutations in *MAGED2*, which promotes expression and activity of salt-transporter NKCC2, cause a similar antenatal phenotype during gestation as those in the gene encoding NKCC2 [6,7,8]. More recently, we demonstrated that MAGED2 is required under hypoxia to allow for the generation of cAMP, which is essential for the cell surface expression and function of NKCC2, thus explaining, at least in part, the salt loss in patients with MAGED2 mutations [9]. The idea that MAGED2 shields renal salt transport from hypoxic stress by allowing cAMP generation needed for NKKC2 expression and function concurs with recent studies demonstrating that many members of the large family of MAGE proteins protect against various forms of stress [10] by modulating activity and substrate specificity of ubiquitin E3 ligases [11]. Ubiquitination denotes a form of posttranslational modification (PTM), which is mediated by enzymes that catalyze ubiquitin activation (E1s), coupling (E2s), and binding to protein targets (E3s), as well as by deubiquitination enzymes, which remove ubiquitin molecules and chains from targets. E3 ubiquitin ligases determine the exact substrate specificity of ubiquitination. Therefore, alterations in E3 activity and subsequent changes in the ubiquitin-proteasome system (UPS), protein quality control, protein trafficking, and other ubiquitin-driven pathways affect all biological processes.

Compensation for fetal hypoxia is of critical importance because it can be aggravated by several internal (maternal anemia, placental insufficiency, umbilical cord pre-eclampsia, cardiac and pulmonary disease) and external factors (smoking 25% of all pregnancies in the United States), exposure to environmental pollutants, and living at high altitude (140 million people worldwide) [1], which may further decrease blood flow to the kidneys [12].

Under normoxia, the transcription factor HIF-1α is hydroxylated by prolyl hydroxylases (PHD), which targets HIF-1α for proteasomal degradation by the E3 ubiquitin Ligase Von Hippel Lindau. Hypoxia inhibits PHD and thereby stabilizes HIF-1α, which promotes the transcription of many genes including erythropoietin and vascular endothelial growth factor (VEGF) [13]. Not surprisingly, HIF-1α and VEGF are expressed abundantly in the fetal renal medulla [4]. Next to prolyl-hydroxylation and ubiquitination, various other types of PTM including phosphorylation [14,15] regulate HIF-1α stability. A role of cAMP-mediated signaling was suggested by studies using β-adrenergic receptor (β-AR) blockade which revealed a markedly diminished induction of erythropoietin to hypoxia in animal models [16,17]. In line with these findings, hypoxia was shown to increase cAMP-dependent protein kinase A (PKA) in various human carcinoma cell lines [18,19]. A recent study demonstrated that PKA activates the transcription HIF-1α gene and that PKA-dependent phosphorylation stabilizes HIF-1α [20].

The aim of the present study is to identify a potential role of MAGED2 in the hypoxic induction of HIF-1α, given that the former is essential for cAMP generation under hypoxia, both of which stabilize HIF-1α expression. We could demonstrate that maximal and appropriate hypoxic induction HIF-1α requires MAGED2 by allowing sufficient cAMP levels. Moreover, we identified PKA Type II as the relevant isoform for this effect. We further revealed that HIF-1α protein is required for normal MAGED2 protein levels and conversely, that MAGED2 expression is stimulated by cAMP mimetics under hypoxia.

Our findings unveil a reciprocal control of MAGED2 and HIF-1α to promote their proper expression under hypoxia, which is mediated by cAMP-dependent induction of PKA type II.

## 2. Materials and Methods

### 2.1. Cell Culture

Human embryonic kidney (HEK293) and HeLa cells (Table 1) were cultured at 37 °C in a humidified environment containing 5% CO_2_ in DMEM Glutmax complemented with 10% fetal bovine serum superior (Sigma-Aldrich, St. Louis, MO, USA), penicillin (100 units/mL), and streptomycin (100 units/mL). For chemical treatment experiments, the media of confluent cells was changed to DMEM serum-free for 14–16 h, Forskolin (10 µM) and Isoproterenol (10 µM) were added to the media at the same time as hypoxia treatment, and the PKA inhibitors, Rp-cAMPS (100 µM) and Rp-8-Br-cAMPS (50 µM) were administered to the cells 30 min before lysis. The control and experimental groups’ cells are always generated from the same flask and passage, and they are studied on the same day.

### 2.2. Cobalt Chloride Treatment

Cobalt chloride (CoCl_2_), a hypoxia mimetic was used to induce HIF-1α in the cells (“chemical hypoxia” [22]). The media of confluent cells was changed to DMEM without serum with 300 µM CoCl_2_ or the desired dose for the dose–response experiment for hypoxia incubation. For 14–16 h, cells were placed in a standard humidified incubator at 37 °C. A Western blot for HIF-1α protein expression was used to assess hypoxia.

### 2.3. Physical Hypoxia

In a modular hypoxia incubator chamber, cells were exposed to physical hypoxia (Billups-Rothenberg, Inc., San Diego, CA, USA; Cat. MIC-101). After the cells had reached ~100% confluence, the media was changed to DMEM without serum, and the cells were put in the center of the chamber, which was sealed shut and linked to a gas tank containing 1% O_2_, 5% CO_2_, and 94% N_2_ through a single flow meter (Billups-Rothenberg, Inc., San Diego, CA, USA; Cat. SFM-3001). The hypoxia chamber was placed in a standard incubator humidified at 37 °C for 14–16 h or the time indicated for the time course experiment. Outside the hypoxia chamber, a normoxic control was placed in the same incubator. A Western blot for HIF-1α protein expression was used to assess hypoxia.

### 2.4. Small Interfering RNA (siRNA) Transfection

Control, MAGE-D2, GNAS, and HIF-1α siRNAs were purchased as ON-TARGETplus SMARTpools from Dharmacon (D-001810-10-05, L-017284-01-0005, L-010825-00-0005, and L-004018-00-0005). By reverse transfection, cells were initially transfected with control or specific siRNA using Lipofectamine RNAiMAX (Invitrogen, Waltham, MA, USA) according to the manufacturer’s instructions.

### 2.5. Western Blotting

Cells were lysed in lysis buffer (50 mM Tris pH 7.4, 5 mM EDTA, 150 mM NaCl, 1 percent Triton X-100, and protease inhibitors) after three washes with ice-cold phosphate-buffered saline (PBS), and cell lysates were cleared at 13,000 g for 15 min at 4 °C. A Pierce^TM^ BCA Protein Assay Kit (Thermo Scientific^TM^, Waltham, MA, USA) was used to determine the protein contents in the supernatants. Proteins were separated on 7.5% TGX Stain Free gels (Bio-rad, Hercules, CA, USA; Cat. 1610181) and transferred to nitrocellulose membranes (Bio-rad, Cat. 1704270) using a Trans-Blot Turbo Transfer System (Bio-rad). Fluorescence antibodies StarBright Blue 520 and 700 were used to identify the proteins (Bio-rad). The blots were imaged with a ChemiDoc MP imaging system (Bio-Rad). ImageJ software was used to determine the gray density of Western blots (National Institutes of Health, Bethesda, MD, USA).

### 2.6. Quantitative Real-Time Reverse Transcription PCR (qRT-PCR)

The mRNA amount of MAGE-D2 was determined by quantitative real-time RT-PCR (qRT-PCR) and the GAPDH gene was used as the internal control. The total RNA of cells was isolated using the SingleShot Cell Lysis Kit (Bio-rad) and transcribed to complementary DNA (cDNA) with the iScript™ Advanced cDNA Synthesis Kit (Bio-rad). PCR was carried out with SsoAdvanced Universal SYBR Green Supermix (Bio-rad) on a 7500 Real-Time PCR System (Applied Biosystems, Waltham, MA, USA). Cycle threshold values were normalized to amplification of GAPDH. 

### 2.7. Statistical Analyses

Results are expressed as mean ± SEM Differences between means were evaluated using unpaired Student *t*-test or two-way ANOVA test. Statistical analyses were performed using GraphPad Prism X9 software. *p* ≤ 0.05 was considered statistically significant (*), *p* ≤ 0.01 was considered highly significant (**), and *p* ≤ 0.001 was considered very highly significant (***).

## 3. Results

### 3.1. MAGED2 Is Required for Hypoxic Induction of HIF-1α

**Figure 1:**
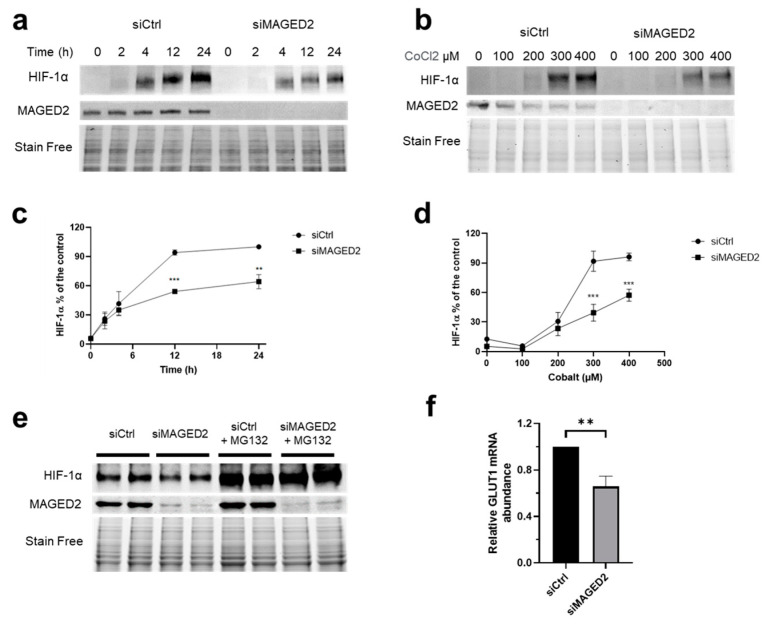
MAGED2 promotes hypoxic HIF-1α protein expression in HeLa cells. HeLa cells were transfected with control (siCtrl), MAGED2 (siMAGED2). Briefly, 24–48 h post-transfection, cells were exposed to physical hypoxia (**a**,**e**) or to the hypoxia mimetic CoCl_2_ (**b**): (**a**) Cells were exposed to physical hypoxia (1% O_2_, 5% CO_2_, 94% N_2_) for the specified times. (**b**) Cells were exposed to CoCl_2_ with the indicated dose of CoCl_2_ for 14–16 h. Total cell lysates were separated by SDS-PAGE and probed with anti-HIF-1α and MAGED2 antibodies. (**c**,**d**) Densitometric analysis of HIF-1α immunoblot is presented in (**a**,**d**), respectively. (**e**) Cells were exposed to physical hypoxia and treated with the proteasome inhibitor, 10 µM MG132. (**f**) Cells were exposed to physical hypoxia for 14 h. Total RNA was extracted, and the relative mRNA amount of GLUT1 was determined by qRT-PCR. Statistical significance was determined by two-way ANOVA test (**c**,**d**) or unpaired two-sided Student’s *t*-test (**f**). All Western blots shown are from the same experiment, which is a representative example of three independent experiments. Bar graphs show mean ± SEM. ** *p* ≤ 0.01 and *** *p* ≤ 0.001.

As activation of the cAMP/PKA pathway stabilizes HIF-1α by phosphorylation and enhances its transcriptional activity [20,23], we analyzed the effect of MAGED2 knockdown on the induction of HIF-1α using physical hypoxia (Figure 1a,c and Figure S1a) or CoCl_2_ (Figure 1b,d) to induce HIF-1α in HeLa cells. As expected, physical hypoxia induced HIF-1α expression in a time-dependent manner (Figure 1a,c and Figure S1a). Interestingly, as can be seen in Figure 1a,c and Figure S1a, MAGED2 knockdown significantly reduced the induction of HIF-1α by physical hypoxia. Likewise, the same effect was observed with CoCl_2_ given that MAGED2 knockdown reduced again HIF-1α expression (Figure 1b,d). As PKA-dependent phosphorylation has been shown to reduce proteasomal degradation of HIF-1α [20], we analyzed the effect of the proteasome inhibitor MG132 on HIF-1α (Figure 1e). We revealed that this compound neutralized the negative effect of MAGDE2 depletion on HIF-1α expression, which supports the notion, that PKA-dependent phosphorylation impairs proteasomal degradation of HIF-1α in MAGED2 depleted cells. We next asked if reduced HIF-1α protein expression translated into decreased mRNA levels of GLUT1, a classical HIF-1α transcriptional target [24]. As shown in Figure 1f, MAGED2 depletion did indeed reduce GLUT1 mRNA abundance.

### 3.2. MAGED2 Is Required for Hypoxic Induction of HIF-1α Independently of the Expression System

**Figure 2:**
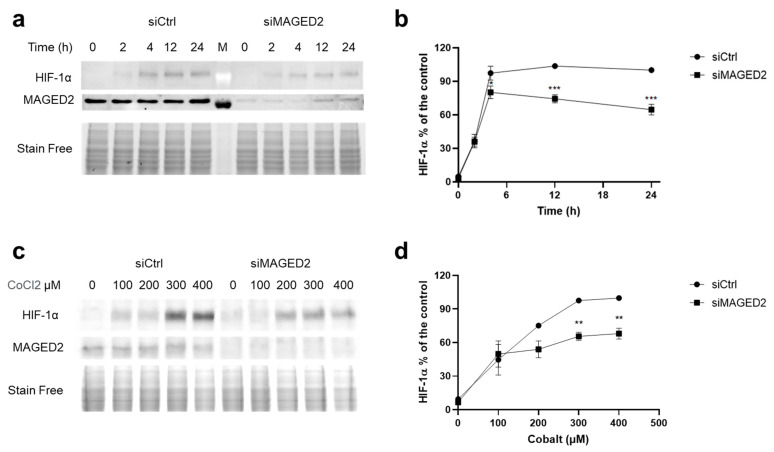
MAGED2 promotes hypoxic HIF-1α protein expression in HEK293 cells. HEK293 cells were transfected with control (siCtrl), and MAGED2 (siMAGED2). Briefly, 24–48 h post-transfection, cells were exposed to physical hypoxia (**a**) or to the hypoxia mimetic CoCl_2_ (**c**): (**a**) Cells were exposed to physical hypoxia (1% O_2_, 5% CO_2_, 94% N_2_) for the specified times. (**c**) Cells were exposed to CoCl_2_ with the indicated dose for 14–16 h. Total cell lysates were separated by SDS-PAGE and probed with anti-HIF-1α and MAGED2 antibodies. (**b**,**d**) Densitometric analysis of HIF-1α immunoblot is presented in (**a**,**c**), respectively. Statistical significance was determined by two-way ANOVA test. All Western blots shown are from the same experiment, which is a representative example of three independent experiments. Bar graphs show mean ± SEM. * *p* ≤ 0.05, ** *p* ≤ 0.01 and *** *p* ≤ 0.001.

As protein regulations and subcellular distributions may be cell-dependent and therefore depend on the expression system used, we sought to confirm our findings by conducting the experiments in another cell line, the HEK293 cells. As illustrated in Figure 2 and Figure S1b, similar to HeLa cells, MAGED2 depletion in HEK293 cells significantly impaired the induction of HIF-1α by physical hypoxia and CoCl_2_. Taken in concert, these data confirm our observation that MAGED2 is required for maximal induction of HIF-1α under hypoxia and clearly indicate that the MAGED2 effect on HIF-1α is independent of the expression system.

### 3.3. Similar to MAGED2, Gαs Is Required for Hypoxic Induction of HIF-1α

**Figure 3:**
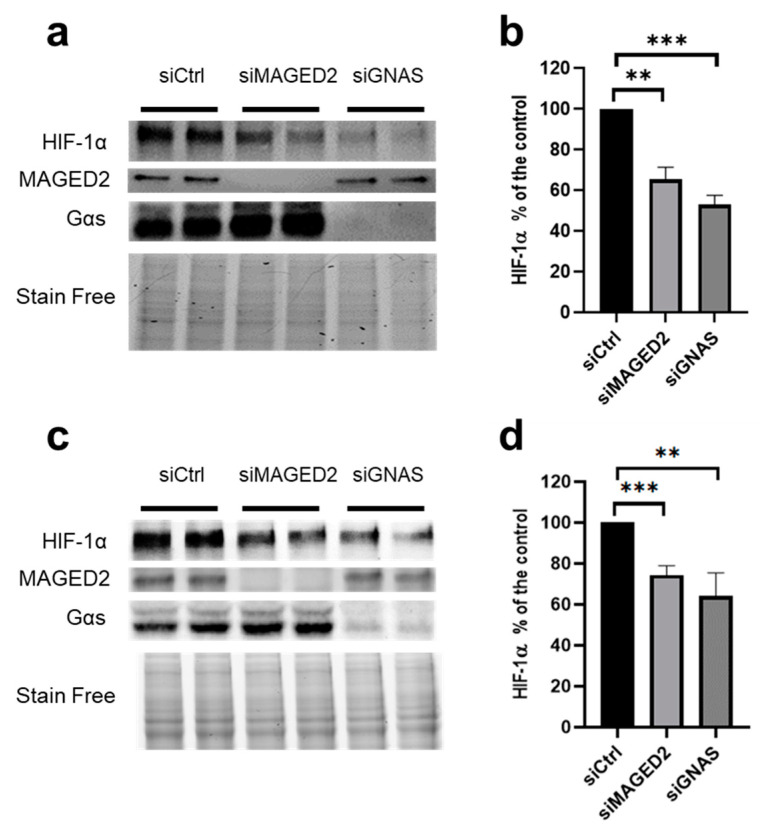
MAGED2 and Gαs promote hypoxic HIF-1α protein expression in HeLa cells. HeLa cells were transfected with control (siCtrl), MAGED2 (siMAGED2) or GNAS (siGNAS) siRNA. Briefly, 24–48 h post-transfection, cells were exposed to physical hypoxia (**a**) or 300µM CoCl2 (**c**) for 14–16 h. Total cell lysates were separated by SDS-PAGE and probed with anti-HIF-1α, MAGED2, and Gαs antibodies. (**b**,**d**) Densitometric analysis of HIF-1α immunoblot is presented in (**a**,**c**). Statistical significance was determined by unpaired two-sided Student’s *t*-test (**b**,**d**). All Western blots shown are from the same experiment, which is a representative example of three independent experiments. Bar graphs show mean ± SEM. ** *p* ≤ 0.01 and *** *p* ≤ 0.001.

We have shown previously that MAGED2 depletion inhibits the functioning of Gαs by causing its MDM2 and ubiquitin-dependent internalization, which precludes activation of membrane-bound adenylate cyclase and hence cAMP generation [9]. To investigate if the effects of the MAGED2 knockdown on HIF-1α expression are indeed caused by inhibiting Gαs, we analyzed also the effect of GNAS knockdown on hypoxic HIF-1α induction by exposing cells to physical hypoxia (Figure 3a,b) or to the hypoxia mimetic CoCl_2_ (Figure 3c,d). Similar to MAGED2 knockdown, Gαs knockdown decreased also the induction of HIF-1α to a comparable degree under both conditions. Thus, these data strongly suggest not only that MAGED2 is required for full and appropriate hypoxic induction of HIF-1α, but also that the positive effect of MAGED2 on HIF-1α protein expression involves the cAMP/PKA pathway initiated upstream by Gαs.

### 3.4. Activation of the cAMP/PKA Pathway Reversed the Effect of MAGED2 Knockdown on Hypoxic HIF-1α Induction

**Figure 4:**
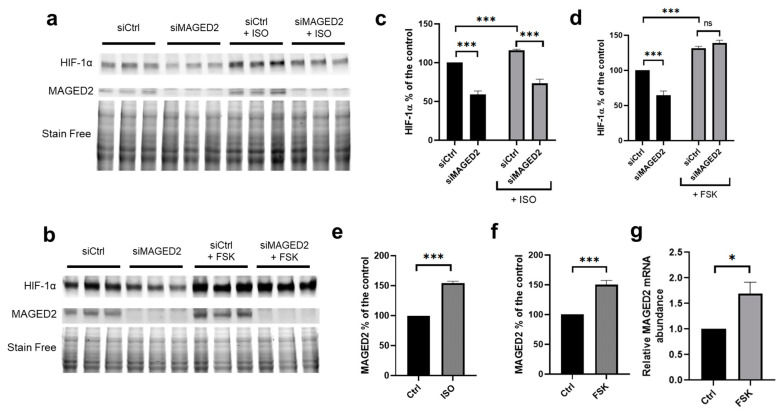
Forskolin reverses the effect of MAGED2 knockdown on hypoxic HIF-1α induction and induces MAGED2 transcription: (**a**,**b**) HeLa cells were transfected with control (siCtrl) or MAGED2 (siMAGED2) siRNA. Cells were exposed to the hypoxia mimetic CoCl_2_ (300 µM) and with (**a**) 10 µM isoproterenol (ISO) or (**b**) 10 µM forskolin (FSK) for 14–16 h. Total cell lysates were separated by SDS-PAGE and probed with anti-HIF-1α and MAGED2 antibodies. (**c**,**d**) Densitometric analysis of HIF-1α immunoblot presented in (**a**,**b**), respectively. (**e**,**f**) Densitometric analysis of MAGED2 immunoblot presented in (**a**,**b**), respectively. (**g**) HEK293 cells were treated with CoCl_2_ for 14 h and then treated with DMSO or Forskolin 10 µM for 2 h. Total RNA was extracted, and the relative mRNA amount of MAGED2 was determined by qRT-PCR. Statistical significance was determined by unpaired two-sided Student’s *t*-test (**c**–**g**). All Western blots shown are from the same experiment, which is a representative example of three independent experiments. Bar graphs show mean ± SEM. * *p* ≤ 0.05 and *** *p* ≤ 0.001.

To independently confirm that MAGED2 acts via Gαs to induce the expression of HIF-1α, we compared the effects of isoproterenol and forskolin on the expression of HIF-1α induced by CoCl_2_ in HeLa cells. Isoproterenol is a β2-adrenergic receptor agonist acting upstream of Gαs, whereas forskolin, is an activator of membrane-bound adenylate cyclase, acting downstream of Gαs. As shown in Figure 4a–d and Figure S3, isoproterenol was unable to reverse the effect of MAGED2 knockdown on HIF-1α protein expression. In contrast, forskolin reversed the impaired induction of HIF-1α caused by MAGED2 knockdown. Together, these results confirm that MAGED2 is essential for promoting the expression of HIF-1α via the cAMP/PKA pathway under hypoxia, and that regulation occurs at the level of Gαs.

As expected, forskolin also reversed the effect of MAGED2 knockdown on HIF-1α protein expression in HEK293 cells (Figure S2).

### 3.5. Activation of the cAMP/PKA Pathway Increased MAGED2 mRNA and Protein Abundance

Interestingly, we noticed that forskolin increased MAGED2 protein levels under hypoxic conditions (Figure 4a,b,e,f). As the MAGED2 promoter contains four CREB1 (CAMP responsive element binding protein 1) binding sites at the positions-122, -1311, -1468, and -1980 relative to the transcription start site (https://epd.epfl.ch//index.php, accessed on 15 July 2019, [25]) we asked if activation of the cAMP/PKA pathway may induce transcription of the MAGED2 gene via enhanced binding of the transcription factor CREB to the four CREB1 sites. To address this question, we analyzed MAGED2 mRNA levels in HEK293 cells exposed to CoCl_2_ and treated with forskolin. As illustrated in Figure 4g, forskolin increased MAGED2 mRNA abundance under hypoxia, which is in agreement with our finding of enhanced MAGED2 protein expression under the same experimental conditions.

### 3.6. PKA type II Regulates the Expression of HIF-1α

**Figure 5:**
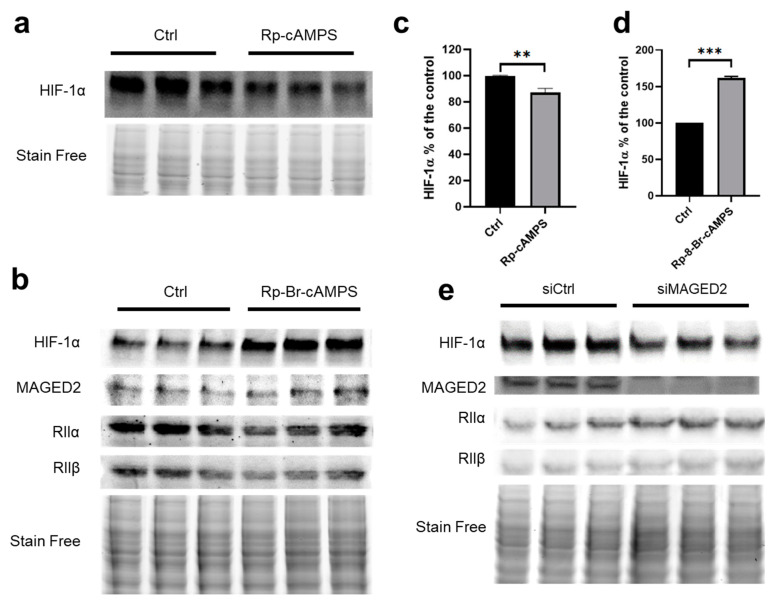
PKA type II regulates HIF-1α protein abundance: (**a**,**b**) HEK293 cells were treated with the non-selective PKA inhibitor (Rp-cAMPS, 100 µM) or the selective PKA type I inhibitor (Rp-8-Br-cAMPS, 50 µM) for 30 min under CoCl_2_. Total cell lysates were separated by SDS-PAGE and probed with anti-HIF-1α, MAGED2, RIIα, or RIIβ antibodies. (**c**,**d**) Densitometric analysis of HIF-1α immunoblot presented in (**a**,**b**), respectively. (**e**) cells were transfected with control (siCtrl) or MAGED2 (siMAGED2) siRNA. Cells were exposed to 300 µM CoCl_2_ for 14–16 h. Total cell lysates were separated by SDS-PAGE and probed with anti-HIF-1α, MAGED2, RIIα, and RIIβ antibodies. Statistical significance was determined by unpaired two-sided Student’s *t*-test (**c**,**d**). All Western blots shown are from the same experiment, which is a representative example of three independent experiments. Bar graphs show mean ± SEM. ** *p* ≤ 0.01 and *** *p* ≤ 0.001.

To determine which type of PKA regulates the expression of HIF-1α under hypoxic conditions, we treated cells with a non-selective PKA inhibitor (Rp-cAMPS, 100 µM) or a selective PKA type I inhibitor (Rp-8-Br-cAMPs, 50 µM). Rp-cAMPS slightly lowered HIF-1α protein abundance (Figure 5a,c). As expected, the selective PKA type I inhibitor Rp-8-Br-cAMPs, which activates PKA type II at 50 µM [26], had the opposite effect and increased HIF-1α expression (Figure 5b,d). Accordingly, we observed a decreased abundance of the regulatory subunits RIIα and RIIβ (Figure 5b), reflecting PKA type II activation. Together, these findings indicate that PKA type II is the PKA isoform involved in the regulation of HIF-1α. In line with these findings, MAGED2 knockdown increased RIIα and RIIβ expression under hypoxic conditions reflecting a decrease in PKA type II activity (Figure 5e).

### 3.7. HIF-1α Promotes MAGED2 Expression under Hypoxia

**Figure 6:**
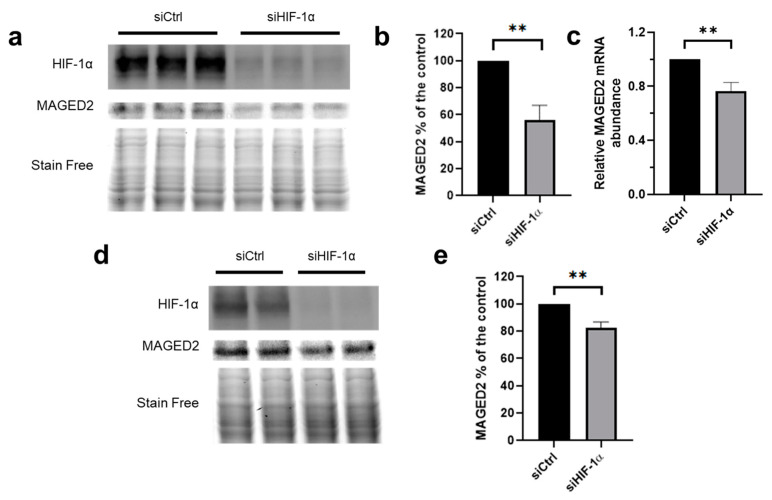
HIF-1α knockdown reduces MAGED2 expression under hypoxia. HeLa (**a**,**c**) and HEK293 (**d**) cells were transfected with control or HIF-1α siRNA. cells were treated with 300 µM CoCl_2_ for 14–16 h: (**a**,**d**) Total cell lysates were separated by SDS-PAGE and probed by anti-HIF-1α and MAGED2 antibodies. (**b**,**e**) densitometric analysis of HIF-1α immunoblot presented in (**a**,**d**), respectively. (**c**) HeLa cells were exposed to physical hypoxia for 14 h. Total RNA was extracted, and the relative mRNA amount of MAGED2 was determined by qRT-PCR. Statistical significance was determined by unpaired two-sided Student’s *t*-test (**b**,**c**,**d**). All Western blots shown are from the same experiment, which is a representative example of three independent experiments. Bar graphs show mean ± SEM. ** *p* ≤ 0.01.

As HIF-1α was previously shown to increase PKA activity under hypoxia [27] and our finding that forskolin increases MAGED2 mRNA levels and protein levels under hypoxia (Figure 4), we hypothesized that HIF-1α is essential for the induction of MAGED2 protein expression. To this end, we analyzed the effect of HIF-1α knockdown under hypoxia in HEK293 and HeLa cells. As illustrated in Figure 6, HIF-1α knockdown decreases MAGED2 protein expression in HeLa (Figure 6a,b) and HEK293 cells (Figure 6d,e) exposed to CoCl_2_ demonstrating that HIF-1α is required for expression of MAGED2 protein under these conditions. Likewise, using physical hypoxia in HIF-1α depleted HeLa cells, a significant reduction of MAGED2 mRNA was observed (Figure 6c).

## 4. Discussion

The key finding of this study is that MAGED2 is required under both physical hypoxia and treatment with the hypoxia mimetic CoCl_2_ to fully induce HIF-1α in renal (HEK293) and cancer (HeLa) cell culture models. Our finding of interdependent regulation of MAGED2 and HIF-1α via PKA type II under hypoxia provides a mechanism for how MAGED2 through induction of HIF-1α promotes cAMP generation, which is essential in the hypoxic fetal kidneys to promote salt reabsorption. Thus, the lack of the MAGED2 and HIF-1α amplification loop compromises cAMP generation, which leads to renal salt-wasting in transient Bartter syndrome, caused by truncating mutations in *MAGED2*. 

Our data with PKA subtype-specific agents indicate that PKA type II is the relevant PKA isoform involved in the regulation of HIF-1α. This notion was further corroborated by the observation that the regulatory subunits RIIα and RIIB (which- together with the catalytic subunits- constitute PKA type II) were increased in hypoxia with the knockdown of MAGED2, (Figure 5e). In agreement with our findings, PKA type II was shown to be expressed in the majority of organs [28,29] whereas PKA type I is mainly expressed in the brain [30]. In addition, the notion that PKA type II is involved in the hypoxic induction of HIF-1α is in line with the study from Lucia and coworkers, who demonstrated that the hypoxic induction of PKA type II is mediated by hypoxic repression of RIIβ gene transcription [27]. 

Of interest, our studies under hypoxia also revealed that isoproterenol and forskolin increased MAGED2 protein abundance and that *GNAS* knockdown elicits the opposite effect. This clearly indicates that the Gαs/cAMP/PKA pathway promotes MAGED2 expression under hypoxic conditions. To further support this notion, we also showed that forskolin increases the abundance of MAGED2 transcript under hypoxic conditions, which is in agreement with the presence of four CRE sites in the 2000 bp of the MAGED2 promoter. The lack of effect of isoproterenol in MAGED2-depleted cells on the hypoxic induction of HIF-1α concurs with our previous study showing that MAGED2 is essential for Gαs dependent activation of membrane-bound adenylate cyclase [9] thereby explaining that agents acting upstream of Gαs such isoproterenol are ineffective in MAGED2-depleted cells. 

**Figure 7:**
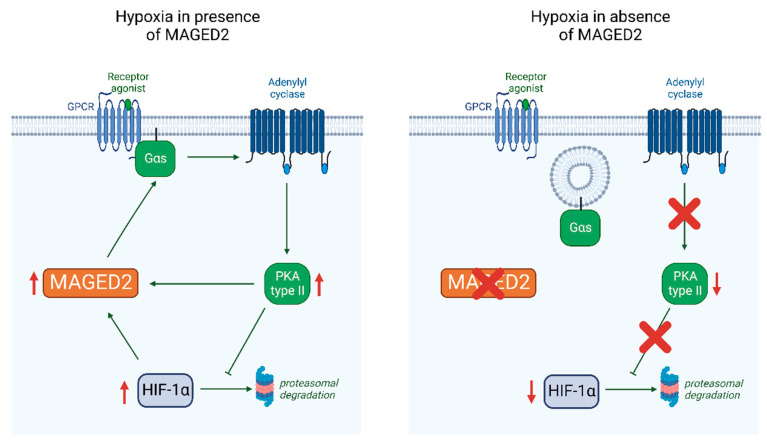
Proposed model for MAGED2′s role under hypoxia (created with BioRender.com). Under hypoxia, MAGED2 inhibits Gαs endocytosis. This ensures activation of the adenylate cyclase and cAMP generation and activation of PKA, which enhances expression of HIF-1α under hypoxia by inhibiting its proteasomal degradation. The latter amplifies together with forskolin MAGED2 mRNA levels. Hence, depletion of MAGED2 impairs the cAMP/PKA pathway and induction of HIF-1α. Reduced cAMP levels explain salt loss in transient Bartter syndrome.

In light of the interdependence between MAGED2, Gαs/cAMP/PKA, and HIF-1α shown in this study we asked if HIF-1α also regulates MAGED2 expression. We could indeed show that HIF-1α was necessary for maximal MAGED2 protein expression. Given that the MAGED2 promoter does not contain HRE elements, which precludes direct activation of the MAGED2 promoter by HIF-1α, we speculate that reduced MAGED2 expression resulted from reduced PKA activity possibly by increased PRKAR2B gene transcription under hypoxic conditions [27], which represses PKA type II activity and hence reduces MAGED2 mRNA levels. To this end, we suggest that under hypoxic conditions, MAGED2 inhibits Gαs endocytosis. This ensures the activation of adenylate cyclase and cAMP and the activation of PKA type II, which enhances HIF-1α expression under hypoxic conditions. The latter increases (similar to forskolin) MAGED2 mRNA levels. Thus, MAGED2 depletion impairs the cAMP/PKA pathway and HIF-1α induction. Decreased cAMP level explains, at least in part, the salt loss in transient Bartter syndrome (Figure 7). This model is further supported by our finding that MAGED2 stabilizes HIF-1α by inhibiting its proteasomal degradation in a PKA-dependent manner. The functional importance of this pathway is demonstrated by reduced GLUT1 mRNA levels, a classical transcriptional target of HIF-1α.

The biological implications of MAGED2’s role in amplifying hypoxic induction of HIF-1α are broad because MAGED2 is expressed in many adult tissues where it could play a critical role under hypoxic conditions (i.e., high altitude, inflamed hypoxic tissue, acute kidney injury, and cancer). Although our study was conducted in cell culture, it is likely that this reciprocal regulation of MAGED2 and HIF-1α, could also take place in native tissues, in particular the kidney, and play a crucial role in the chronic renal adaptations to physiological or pathological challenges: Of note, MAGED2 protein is constitutively expressed in the distal tubule [8,31] while HIF-1α protein is absent in adult kidney, both are induced in the distal tubule of the kidney in rodent models of acute kidney injury (AKI) [31,32,33]. HIF-1α protein has also been demonstrated in humans with renal transplant failure [34]. In conjunction with our data, it is conceivable that MAGED2 is required for proper induction of HIF-1α in the distal tubule. As several research groups have demonstrated a protective effect in animal models of AKI by exogenous induction of HIF1 either by small molecules or by genetic techniques (Table 2 in [32]), activation of MAGED2 might be an additional route to increase HIF-1α activity. Moreover, we previously showed that MAGED2 mutations cause transient Bartter syndrome characterized by severe renal salt wasting and polyuria in fetuses, by heavily altering the expression of two critical renal salt-transporters, NKCC2, and NCC. It is also conceivable that physiological or pathological changes in the expression level of HIF-1α and/or MAGED2 may affect the activity of NKCC2 and/or NCC, therefore altering the water balance and sodium homeostasis, not only during pregnancy but also during adult life. 

In regard to the transient nature of aBS caused by MAGED2 mutations (tBS5), which denotes spontaneous recovery from salt-wasting in parallel with a developmental increase in renal oxygenation, we hypothesized that MAGED2 function is required mainly under physiological hypoxic conditions such as in fetuses. Given the well-established link between HIF-1α and cAMP/PKA and by showing in the present study that MAGED2 is also required for hypoxic induction of HIF-1α, the finding of the present study may further explain the transient nature of this kidney disease. However, the effect of MAGED2 on cAMP and HIF-1α pathways, may still not entirely explain the profound salt wasting caused by impaired NKCC2 and NCC post-Golgi regulation and targeting to the cell surface. Indeed, one cannot exclude that, in MAGED2-depleted hypoxic cells, other molecular mechanisms such as ER retention and associated degradation of the cotransporters due to ER stress induced by hypoxia, are also involved. In line with this idea, we previously showed that export from the ER constitutes the limiting step in NKCC2 maturation and cell surface expression and that WT NKCC2 and its disease-causing mutants are subject to regulation by endoplasmic reticulum-associated degradation (ERAD), in particular under ER stress conditions [35,36,37,38,39].

Importantly, our previous studies showed that MAGED2 depletion impaired the maturation of the transiently expressed key renal transport proteins and membrane proteins NKCC2 and NCC in HEK293 cells [8]. Given that transient overexpression induces ER stress [40], especially when overexpressing transmembrane proteins [41], we speculate that this stress rendered the maturation of NKCC2 and NCC MAGED2 sensitive. Along these lines, it is conceivable that the effects of MAGED2 depletion on HIF-1*α* induction and G*α*s localization seen with cobalt chloride results from redox stress [9]. Taken together, we speculate that MAGED2 protects against various forms of stress, namely hypoxic stress, ER stress, and redox stress. 

MAGED2 is also expressed in many human cancers and is associated with a poor prognosis [4,42,43,44,45]. Moreover, the hypoxic microenvironment in cancer cells is the key condition affecting the cellular expression program leading to chemotherapy resistance [46]. Given the established roles of Gαs and HIF-1α as oncoproteins in malignancy [47,48,49], it is conceivable that MAGED2 promotes tumorigenesis by stimulating the cAMP/PKA- HIF-1α pathway. 

Our data significantly extend previous studies, which uncovered unidirectional links between cAMP/PKA and HIF-1α and vice versa but not their reciprocal interactions governed by MAGED2 acting as a master regulator: Starting with the finding that β-blockers reduce expression of the HIF-1α dependent gene erythropoietin more than 40 years ago, various groups reported unidirectional connections between hypoxia, HIF-1α, and the cAMP/PKA pathway. In a sequential order starting at the plasma membrane, all these studies identified the following links between the HIF-1α and the cAMP/PKA pathways: A hypoxia-specific phosphorylation of the β-receptor is essential for HIF-1α activation [50], adenylyl cyclase VI and VII are induced by HIF-1α [19], hypoxia increases PKA activity [18,27], and PKA stabilizes HIF-1α by phosphorylation [20,23]. Our study now adds substantially to the above findings because we identified MAGED2 as a master regulator, which is connected to HIF-1α and the cAMP/PKA pathways in a multidirectional fashion through multiple and partly independent ways. 

In summary, we identified that MAGED2 acts as a master regulator for the hypoxic induction of HIF-1α by controlling Gαs dependent activation of PKA type II. In terms of therapeutic applications, inhibition of MAGED2 could target the oncoproteins Gαs and HIF-1α specifically in hypoxic tumors, and its activation may enhance HIF-1α induction in kidney disease.

## Figures and Tables

**Table 1:** Reagents and tools.

Reagent or Resource	Source	Identifier
**Antibodies**		
Anti-HIF-1α rabbit	Cell Signaling	14179
Anti-MAGED2 rabbit	This paper	
Anti-RIIβ rabbit	Thermo Fisher Scientific	PA582348
Anti-RIIα mouse	Thermo Fisher Scientific	TA501145
Anti-Gαs	Sigma Aldrich	06-237
StarBright Blue 520 Goat Anti-Rabbit IgG	Bio-rad	12005869
StarBright Blue 700 Goat Anti-Mouse IgG	Bio-rad	12004158
**Chemicals, Peptides, and Recombinant Proteins**		
Forskolin	Sigma-Aldrich	F6886-10MG
(−)-Isoproterenol hydrochloride	Sigma-Aldrich	I6504-100MG
Rp-cAMPS	Sigma-Aldrich	116814-5UMOL
Rp-8-Br-cAMPS	Sigma-Aldrich	116816-5UMOL
**Critical Commercial Assays**		
SingleShot Cell Lysis Kit	Bio-rad	1725080
iScript Advanced cDNA Synthesis Kit for RT-qPCR	Bio-rad	1725038
SsoAdvanced Universal SYBR Green Supermix	Bio-rad	1725271
**Experimental Models: Cell Lines**		
HEK293	ATCC	CRL1573
HeLa	Gift from Dr. Vijay Renigunta	
**Oligonucleotides**		
ON-TARGETplus Non-targeting Control Pool	Dharmacon	D-001810-10-05
UGGUUUACAUGUCGACUAA		
UGGUUUACAUGUUGUGUGA		
UGGUUUACAUGUUUUCUGA		
UGGUUUACAUGUUUUCCUA		
ON-TARGETplus Human MAGED2 siRNA—SMARTpool	Dharmacon	L-017284-01-0005
GGACGAAGCUGAUAUCGGA		
GCUAAAGACCAGACGAAGA		
AGGCGAUGGAAGCGGAUUU		
GAAAAGGACAGUAGCUCGA		
ON-TARGETplus Human GNAS siRNA—SMARTpool	Dharmacon	L-010825-00-0005
GCAAGUGGAUCCAGUGCUU		
GCAUGCACCUUCGUCAGUA		
AUGAGGAUCCUGCAUGUUA		
CAACCAAAGUGCAGGACAU		
ON-TARGETplus Human HIF-1α siRNA-SMARTpool	Dharmacon	L-004018-00-0005
GAACAAAUACAUGGGAUUA		
AGAAUGAAGUGUACCCUAA		
GAUGGAAGCACUAGACAAA		
CAAGUAGCCUCUUUGACAA		
GAPD, Human GAPDH, Real-Time PCR Primer Set	Biomol	VHPS-3541
GAGTCAACGGATTTGGTCGT		
TTGATTITGGAGGGATCTCG		
MAGED2, Human melanoma antigen family D, 2, Real-Time PCR Primer Set	Biomol	VHPS-5486
TTTTGGCTAAAGACCAGACG		
AATAGCCTGCTCGTTCAATG		
GLUT1, Real-Time PCR Primer Set	Sigma-Aldrich	[21]
TCACTGTGCTCCTGGTTCTG		
CCTGTGCTGAGAGATCC		
**Software and Algorithms**		
ImageJ	Schneider et al., 2012	https://imagej.nih.gov/ij/
GraphPad Prism 9	GraphPad	
EndNote X9	Clarivate Analytics	

## Data Availability

All data are available in the main text or the Supplementary Materials.

## Supplementary Materials

The following supporting information can be downloaded at: https://www.mdpi.com/article/10.3390/cells11213424/s1.
