# Supplementary material for: Reciprocal Regulation of MAGED2 and HIF-1α Augments Their Expression under Hypoxia: Role of cAMP and PKA Type II
Source: Cells. 2022 Oct 29;11(21):3424. doi: 10.3390/cells11213424 (PMC9655371; doi:10.3390/cells11213424)
Supplement: Supplementary file 1 [file cells-11-03424-s001.zip › cells-1915356-supplementary.pdf]

Supplementary figures

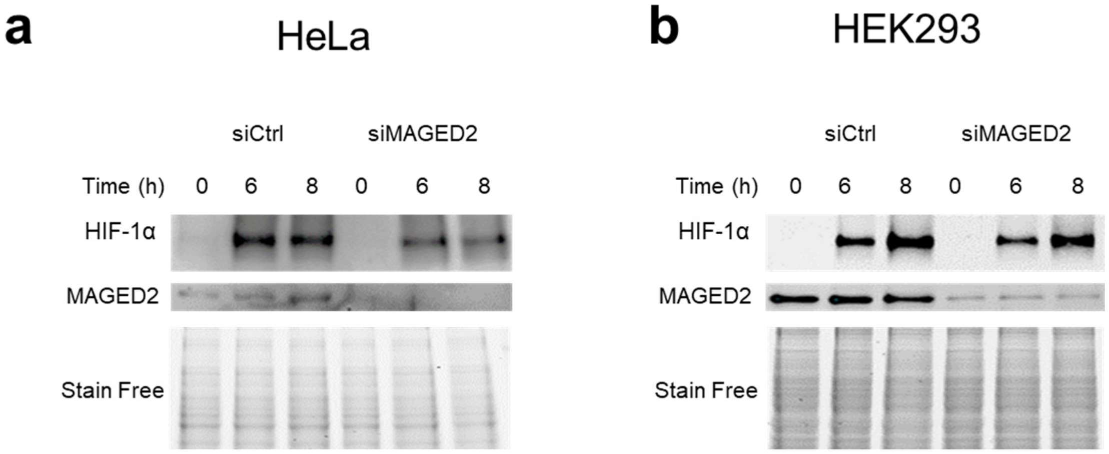

Figure S1. MAGED2 promotes hypoxic HIF-1α protein expression in HeLa and HEK293 cells: HeLa (a) and HEK293 (b) cells were transfected with control (siCtrl), MAGED2 (siMAGED2). Briefly, 24 - 48 h post-transfection, cells were exposed to physical hypoxia. Cells were exposed to physical hypoxia (1% O2, 5% CO2, 94% N2) for the specified times. Total cell lysates were separated by SDS-PAGE and probed with an-ti-HIF-1α and MAGED2 antibodies.

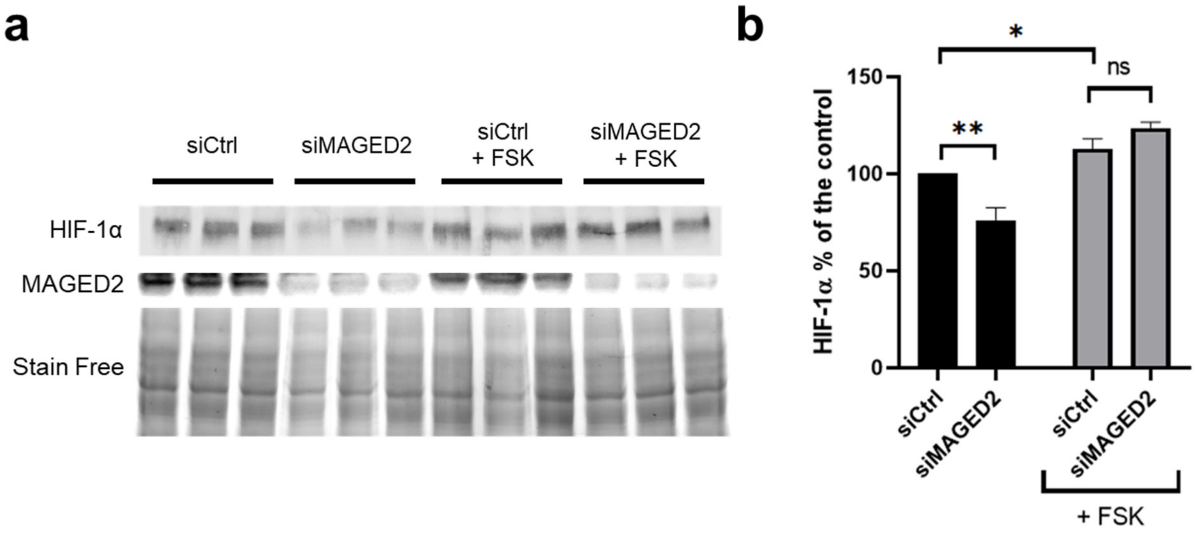

Figure S2. Forskolin reverses the effect of MAGED2 knock-down on hypoxic HIF-1α induction in HEK293 cells: (a) HEK293 cells were transfected with control, or MAGED2 (M) siRNA cells were treated with chemical hypoxia (300μM $CoCl_2$) with 10μM forskolin (FSK) for 14 - 16 h. Total cell lysates were separated by SDS-PAGE and probed with anti-HIF-1α and MAGED2 antibodies. (b) Densitometric analysis of HIF-1α immunoblot is presented in (a). Statistical significance was determined by unpaired two-sided Student's t-tests (b). Bar graphs show mean ± SEM. *P ≤ 0.05 and ** P ≤ 0.01.

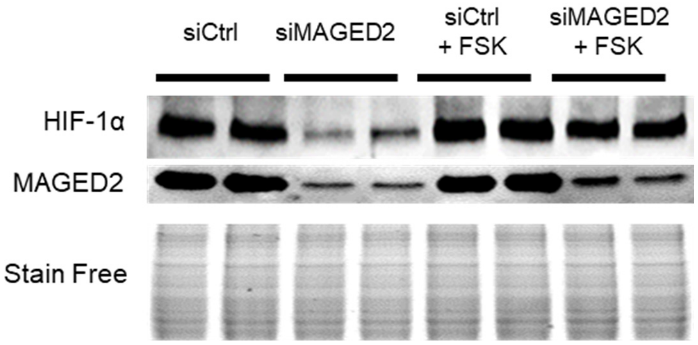

Figure S3. Forskolin reverses the effect of MAGED2 knockdown on hypoxic HIF-1α induction. HeLa cells were transfected with control (siCtrl) or MAGED2 (siMAGED2) siRNA. Cells were exposed to physical hypoxia and with 10μM forskolin (FSK) for 14 - 16 h. Total cell lysates were separated by SDS–PAGE and probed with anti-HIF-1α and MAGED2 antibodies.
